# Teaching a graduate level course in automatic chemical analysis

**DOI:** 10.1155/S1463924679000390

**Published:** 1979

**Authors:** Richard F. Browner

**Affiliations:** School of Chemistry Georgia Institute of Technology Atlanta Georgia 30332 USA


### Teaching a graduate level course in automatic chemical analysis*

Richard F. Browner,

*School of Chemistry, Georgia Institute of Technology, Atlanta, Georgia 30332, USA.*

The traditional attitude of the educator in dealing with automatic chemical analysis has been to place the discussion firmly in the context of instrumentation and electronics. However, many analytical chemistry students graduating today will be faced early in their careers with the need to make managerial decisions based on economic as well as technical factors. With a purely technically based course as background the student may be poorly prepared to consider automation in this broader context. One alternative is to offer a course in which a possible framework for later professional decision making is outlined and both the technical and economic factors that must be considered in this context are discussed. Clearly, in a course of this type, automation is approached very differently compared to a course emphasizing electronics and computer interfacing. For example, strong emphasis is placed on factors such as cost, reliability and the level of operator skill required to run equipment, rather than on electronic circuit components.

Ideally, the educational results of such a course are (1) to alert students to the potential of automatic procedures in analytical chemistry and (2) to motivate them to implement these procedures, whenever appropriate, in their later careers.

The impetus to teach such a course comes from the author's experience in a large government laboratory, prior to entering academic life. In this laboratory one division, the Automation and Computing Division, has the responsibility for advising on the implementation of automatic analytical procedures throughout the laboratory and, where necessary, also for the design, construction and commissioning of specialized automatic equipment. It is from this environment and from the consequent exposure to the whole gamut of technical, economic and political factors that exist when automatic procedures replace established manual procedures, that the background to the current course was derived.

*This paper is based in part on material presented at the Symposium on Automatic Chemical Analysis at the Pittsburgh Conference, 1978.*

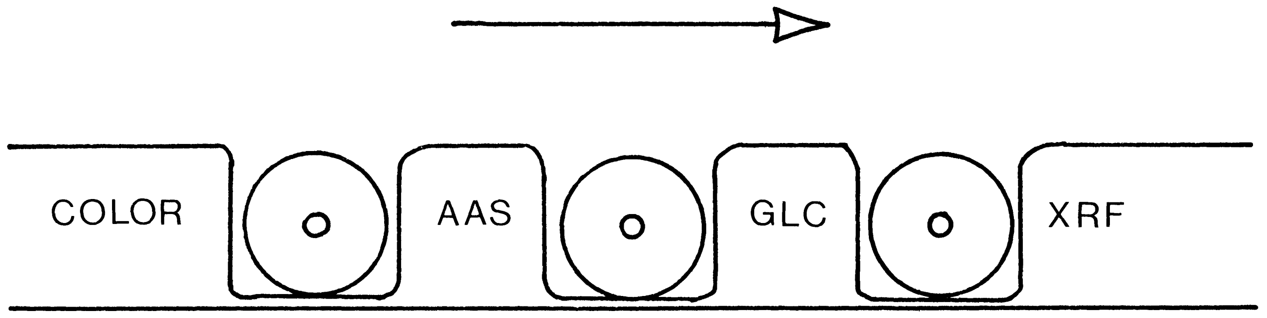

*Figure 1. "Pedagogic peristalsis", or plug flow of information*

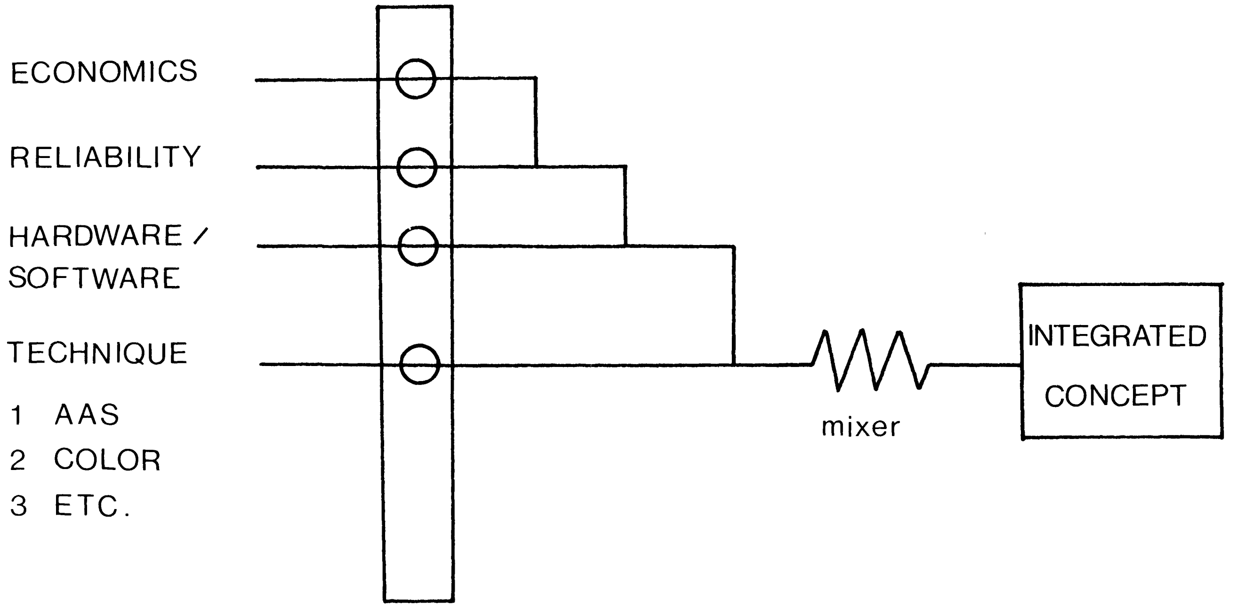

*Figure 2. Integrated approach to teaching of automatic chemical analysis*

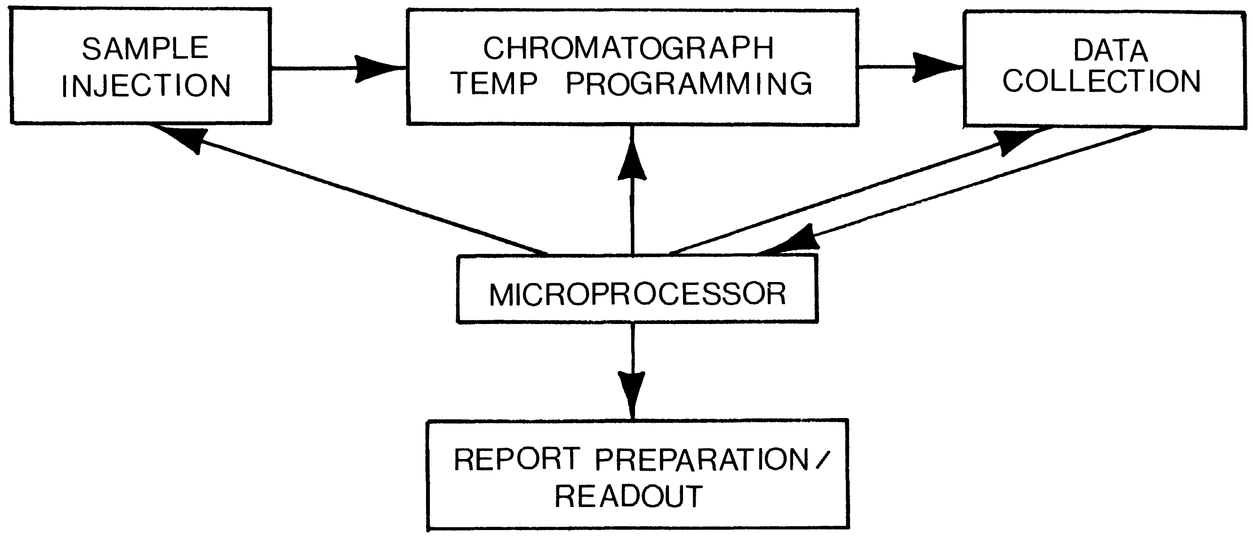

*Figure 3. Automatic control of a gas liquid chromatograph*

It is hoped that the brief outline of course structure which follows, including a more detailed discussion of one selected topic, may be of some value to others in the field.

### Course structure

"Chemistry 8211 - Automatic Chemical Analysis" is a one quarter (10-week) course offered bienially at Georgia Tech. There are three one-hour lectures per week but no laboratory component. During the quarter at least one half-day visit is paid to a large federal laboratory in the area where much of the specialized (and very expensive) automatic equipment described in the course is in regular use. The equipment seen in operation includes Technicon AutoAnalyzers and du Pont ACA and GeMSAEC systems, in addition to automatic gas and liquid chromatographs. Evaluation of student performance is by one-hour midterm "open book" and final three-hour "closed-book" examinations. The exams contain a mixture of essay, short note and factual questions. Student reaction to the course is gauged by a standardized computer read evaluation form and by verbal comments.

The topics covered in the course are:

1. Economic approach
2. Sample handling
3. Continuous automatic analyzers
4. Discrete automatic analyzers
5. Colorimetric detectors
6. Electrochemical detectors
7. Automatic spectrometers (uv/vis and atomic absorption)
8. Gas and liquid chromatographs
9. Thin layer and paper chromatographs
10. Ion exchange chromatographs
11. Solvent extraction
12. Thermal methods
13 Radiometric and x-ray equipment
14. Computer networks and hierarchy
15. Systems approach to laboratory automation

The overlap and cross-referencing inherent in such a course are found to be very beneficial as they emphasize the inter-relationships between various topics. For example, the concepts developed for continuous or discrete automatic analyzers can be applied to areas outside their original clinical chemically oriented purpose and examples are taken frequently to show how innovation of this type can be productive. One example cited in the course is the development of an automated method for the determination of arsenic, sellenium and tellurium in water. The method uses conventional AutoAnalyzer methodology with the incorporation of the development of nascent hydrogen in situ at the manifold to convert the element into its hydrides. The gas stream is passed into a modified atomic absorption spectrometer and quantified. The control exercised in the automatic regime enables the automatic method to offer significant improvements with respect to precision and limit of detection in comparison to a manual approach [1].

The reference text used for the course is "Automatic Chemical Analysis", by J.K. Foreman and P.B. Stockwell [2]. In such a rapidly changing field, new material must be added constantly to reflect the current state-of-the-art, such as for example the current developments in microprocessor technology, but the framework and most of the contents of the book are still valid and useful.

### Avoiding "pedagogical peristalsis"

In a course of this type, it is important to present each topic in context and not as a self-contained 'plug' of information. Such an approach could be represented graphically (Figure 1) as analagous to the peristaltic pumping of information. In the teaching context, the cross-mixing of topics is desirable and an approach closer to that shown in Figure 2 may be more favourable. In this approach, specific topics (such as colorimetry, atomic absorption spectrometry, etc.) are discussed together with a steady admixture of practical features such as economics, reliability of equipment and choice of appropriate automatic approach.

In order for the course to give maximum benefit to students, it helps to keep in mind the likely work situation with which they may be involved later. The most likely areas are (1) industrial, (2) governmental, (3) medical, and (4) academic. In an industrial or government situation, the analytical chemist may function in either an R and D or a management position. In R and D the primary concern will be to develop new analytical procedures, whereas in a more managerial position there will be a greater need to assess the overall role of automation in the laboratory. In medical or health-related environments, automation may be encountered primarily through the implementation of new automatic clinical procedures where co-ordinated record keeping can be of vital concern. In an academic environment, automation of techniques may form part of a research programme. In other words, there can be a very varied emphasis placed by the student upon the material presented in the course, depending on personal career objectives and interests. This makes a wide coverage all important.

### Introduction to the course

Before considering each topic area in detail, a broad survey of possible objectives of automatic analysis is described, taking care that a balanced picture is presented and that possible problems are not ignored. The role of automatic analysis in improvement of data quantity, data quality and economic efficiency is described, considering such aspects as equipment reliability over many hours of unattended operation and self-testing procedures.

Decision points for the implementation of automatic procedures are discussed, namely:

1. Is the commercial equipment currently available suitable for the job at hand?
2. If not, can commercially available equipment be modified to perform the necessary functions?
3. If neither (1) nor (2) apply, can R and D costs for new equipment be justified in cost/benefit terms?

Along with the last point, the possibility of commercial exploitation of novel instrumentation can be considered.

### Specific topics

Three general themes are developed as each topic is considered in turn:

1. The suitability of discrete versus continuous systems
2. Consideration of the cost/benefit of hardwired electronic control versus software implementation of the necessary operating functions
3. Reliability of the optimum automatic procedures compared to existing manual procedures.

To illustrate with a specific example, the approach to gas liquid chromatography will be considered in outline. The components of a gas liquid chromatograph capable of automatic control are first discussed (Figure 3). These include sample injection, oven control (including temperature programming) and more specialized features such as valve control in backflushing or heart cutting. The interactive role of a microprocessor-based control/data collection system is described together with the various degrees of sophistication open to such a system. Each component of the system is then discussed in more detail and possible alternatives considered from economic and other viewpoints. For example, in the case cited, does the flexibility of a microprocessor-controlled sample injection/carousel system outweigh the possible disadvantages of higher cost and maybe lower reliability? How do these factors apply in comparison with a mechanically operated system? Other factors to be considered include

1. The degree of operator skill necessary to ensure reliable operation
2. The context in which extreme flexibility of operation (e.g. variable sample injection volume, out-of-sequence injection of samples) is worthwhile economically
3. The complexity or otherwise of the sample load and how this might affect the choice of equipment
4. The type of data storage and presentation required.

Factors in the last category would include a possible need for recorder traces to provide backup or indicate instrument malfunctions, the required form of data presentation and the possible need for a direct link to a larger computer for storage or sophisticated statistical treatment of data. All of these factors provide decision-making criteria and it is emphasized that there is no single optimum system appropriate to all situations.

Finally, a general discussion relating to the implementation of any automatic procedure as a replacement for an existing manual or semi-automatic procedure is given. Here, the importance of reliability is again emphasized but most emphasis is given to the problems which may be generated by necessary re-organization of the laboratory, re-allocation of personnel and retraining of existing personnel.

### Summary

The essential components of the course in Automatic Chemical Analysis which is offered by the author at the Georgia Institute of Technology have been described briefly. This course probably differs considerably in its emphasis from many others which cover the same topic, mostly by the weight given to economic and managerial considerations. While there is clearly no 'right' approach to this topic, it is hoped that the factors considered in this paper may be seen to offer an alternative and worthwhile approach to the more usual instrumental course coverage.

REFERENCES

[1] Goulden, P.D., and Brooksbank, P., *Analytical Chemistry,* 1974, 46 (11), 1431.

[2] Foreman, J.K., and Stockwell, P.B., "Automatic Chemical Analysis" 1975, Ellis Horwood (Wiley), Chichester, U.K.

